# Patient characteristics and pharmacologic treatment patterns in antifibrotic-treated patients with fibrosing interstitial lung diseases: real-world results from a claims database

**DOI:** 10.1186/s12890-025-03713-x

**Published:** 2025-05-22

**Authors:** Yasuhiro Kondoh, Tomohiro Ito, Hana Kimura, Haikun Bao, Masataka Kuwana

**Affiliations:** 1 Department of Respiratory Medicine and Allergology, Aichi Medical University, 1-1 Yazako Karimata, Nagakute-Shi, Aichi, 480-1195 Japan; 2 Nippon Boehringer Ingelheim Co., Ltd, 2-1-1 Osaki, Shinagawa-Ku, Tokyo, 141-6017 Japan; 3 Boehringer Ingelheim Pharmaceuticals, Inc, 900 Ridgebury Road, Ridgefield, CT 06877 USA; 4 Department of Allergy and Rheumatology, Nippon Medical School Graduate School of Medicine, 1-1-5 Sendagi, Bunkyo-Ku, Tokyo, 113-8602 Japan

**Keywords:** Antifibrotics, Claims data, Database, Japan, Nintedanib, Progression, Pulmonary fibrosis, Real-world data, Post-marketing

## Abstract

**Background:**

Antifibrotics have been approved for use in many countries, including Japan, based on the results of several phase III clinical trials in patients with IPF, SSc-ILD, and PPF, which showed slower lung function decline with antifibrotic treatment. There is a paucity of information on the real-world use of antifibrotics in clinical practice.

**Methods:**

Baseline characteristics, comorbidities, and drugs used prior to and concomitant with antifibrotics were collected for patients with IPF, SSc-ILD, and PPF using a health insurance claims database in Japan from 1 January 2013 to 30 June 2023. Descriptive statistics were generated for all study variables.

**Results:**

This study included 657 nintedanib users with IPF; 418 pirfenidone users with IPF; 4160 nintedanib users with PPF; 18,403 users of glucocorticoids/immunosuppressants for ILD treatment with PPF; 676 nintedanib users with SSc-ILD; and 698 users of glucocorticoids/immunosuppressants for ILD treatment with SSc-ILD. At index, pirfenidone users with IPF were the oldest (mean [SD] 74.8 [7.3] years), and nintedanib users with SSc-ILD were the youngest (mean [SD] 65.6 [11.7] years). In nintedanib users with IPF, 76.7% were prescribed nintedanib as monotherapy, and 75.6% of pirfenidone users were prescribed pirfenidone, as monotherapy. In patients with IPF, 75.2% were prescribed nintedanib, and 76.1% were prescribed pirfenidone, as first-line therapy. In patients with SSc-ILD, 34.9% were prescribed nintedanib as monotherapy for ILD treatment, and 38.6% as first-line therapy. Approximately half of patients with PPF were prescribed nintedanib concomitantly with other glucocorticoids/immunosuppressant drugs, and after one or more glucocorticoids/immunosuppressant drugs. The most common concomitant drug in all patient groups was glucocorticoids. In patients with IPF, 18.6% of nintedanib users and 18.2% of pirfenidone users were prescribed glucocorticoids concomitantly. Concomitant glucocorticoid use was 52.7% for nintedanib users with SSc-ILD, and 44.1% for nintedanib users with PPF.

**Conclusions:**

These results provide real-world evidence of antifibrotic use in clinical practice. Most patients with IPF were prescribed antifibrotics as monotherapy for ILD treatment whereas antifibrotics were used concomitantly with glucocorticoids/immunosuppressants in many patients with SSc-ILD and PPF. While most patients with IPF were prescribed antifibrotics as first-line therapy, patients with SSc-ILD and PPF were more likely to be prescribed nintedanib as second-line or later-line treatment after glucocorticoids/immunosuppressants.

**Supplementary Information:**

The online version contains supplementary material available at 10.1186/s12890-025-03713-x.

## Background

Interstitial lung disease (ILD) is a collective term that encompasses a large heterogenous group of over 200, relatively common to very rare, parenchymal pulmonary disorders [1]. It is characterised by varying degrees of fibrosis, inflammation, or both, in the lung parenchyma [1]. Idiopathic pulmonary fibrosis (IPF) is the most frequent and serious form of fibrosing ILD, with a median survival period of 3–5 years [2]. A proportion of patients with ILDs other than IPF develop a progressive phenotype, termed progressive pulmonary fibrosis (PPF), previously called progressive fibrosing-ILD (PF-ILD) [3, 4]. PPF occurs frequently as a result of autoimmune disease and includes rheumatoid arthritis-associated ILD (RA-ILD), systemic sclerosis-associated-ILD (SSc-ILD), polymyositis/dermatomyositis-associated ILD (PM/DM-ILD), Sjögren’s disease-associated ILD, systemic lupus erythematosus-associated ILD (SLE-ILD), and mixed connective tissue disease-associated ILD (MCTD-ILD), however, there are also non–autoimmune-related ILDs such as chronic hypersensitivity pneumonitis (HP), exposure-related ILD (excluding HP), idiopathic non-specific interstitial pneumonia (iNSIP), unclassifiable idiopathic interstitial pneumonia (IIP), and sarcoidosis [4].

Several phase III trials of antifibrotics in patients with IPF, SSc-ILD, and PPF have shown that pirfenidone and nintedanib slow lung function decline [5–9]. Based on the results of these trials, pirfenidone and nintedanib were approved for the treatment of IPF in the United States, European Union and Japan [10–15], with nintedanib also approved for the treatment of SSc-ILD and PF-ILD in the United States, European Union and Japan [16–20]. The 2015 Official ATS/ERS/JRS/ALAT Clinical Practice Guideline conditionally recommended clinical use of pirfenidone and nintedanib for the treatment of IPF [21]. Recently, 2023 ACR/CHEST guidelines conditionally recommended nintedanib for people with systemic autoimmune rheumatic diseases (SARDs) as a treatment option for SARD-ILD progression despite first-line ILD treatment [22]. Additionally, the 2023 Official American Thoracic Society Clinical Practice Guideline suggests the use of nintedanib, and the combination of nintedanib plus mycophenolate, for the treatment of patients with SSc-ILD [23]. Furthermore, ATS/ERS/JRS/ALAT guidelines conditionally recommend nintedanib treatment for patients with PPF who have failed standard management for fibrotic ILD other than IPF [4]. The Japanese guidelines recommend the use of pirfenidone as a treatment option for IPF, as well as nintedanib as a treatment option for IPF, IIPs (including iNSIP), and subgroups of connective tissue disease-associated ILDs (CTD-ILDs). However, they only provide a treatment algorithm that includes nintedanib for SSc-ILD [24–26]. There is also a paucity of data on patient characteristics and the use of antifibrotics in Japan as well as globally. Two post-marketing surveillance studies are evaluating the safety of nintedanib and its long-term use in clinical practice for SSc-ILD (ClinicalTrials.gov: NCT04325217) and PPF (ClinicalTrials.gov: NCT04559581) in Japan, however, they are both still ongoing, with the PPF study only including a relatively small number of patients with each disease.

The aim of this observational and non-interventional study using the Medical Data Vision (MDV) database, one of the major health insurance claims databases in Japan, was to identify patient characteristics and treatment patterns of antifibrotics in patients with IPF, SSc-ILD, and PPF treated with antifibrotics in real-world clinical practice in Japan. The MDV database was chosen for this study as it contains anonymised administrative claims and Diagnosis Procedure Combination (the Japanese casemix system which is applied in acute hospitals) data for the inpatient and outpatient settings for > 43 million patients from > 400 acute care hospitals in Japan [27]. These hospitals are mainly considered as specialized treatment hospitals. The database collects > 1 million health claims every month, enabling it to provide a broad overview of the real-world disease and treatment landscape in Japan.

## Methods

### Data source

De-identified data from the MDV database from 1 January 2013 through to 30 June 2023 were used, with each patient observed from their first to last encounter in the database. The index date was defined as the first prescription date of the drug of interest. If patients were prescribed more than one drug of interest on different dates or the same date, they were included in both groups.

### Patient information

As nintedanib is currently an approved treatment for IPF, SSc-ILD and PF-ILD, the patient groups of interest were: (i) patients with IPF treated with pirfenidone or nintedanib on or after 31 August 2015 (i.e., the date nintedanib became commercially available), (ii) patients with SSc-ILD treated with nintedanib or oral and IV glucocorticoids/immunosuppressive drugs/disease-modifying anti-rheumatic drugs (DMARDs) for ILD treatment on or after 20 December 2019 (i.e., the approval date of nintedanib for SSc-ILD), and (iii) patients with PPF treated with nintedanib or oral and IV glucocorticoids/immunosuppressive drugs/DMARDs for ILD treatment on or after 29 May 2020 (i.e., the approval date of nintedanib for PF-ILD). The index date is defined as the date of first use of the drug in question i.e., the index date for "nintedanib users in patients with PPF" was the date of first nintedanib use, and the index date of "users of glucocorticoids/immunosuppressants for ILD treatment in patients with PPF" was the date of first use of other drug for ILD treatment. Patients aged ≥ 20 years on the index date were identified based on the inclusion of ILD-related codes of interest, and the absence of flags for only suspected ILD, within 6 months before or on the index date (Supplementary Table S1), which were also used in a previous database study [28]. Patients were treated with antifibrotics or oral and IV glucocorticoids/immunosuppressive drugs/DMARDs for ILD treatment (Supplementary Table S2). All patients must have had extractable baseline data for the 6 months prior to the index date and for ≥ 2 months after the index date for follow-up (Supplementary Figure S1). Excluding IPF patients prescribed pirfenidone before 31 August 2015, SSc-ILD patients prescribed nintedanib/other drugs before 20 December 2019, and PPF patients treated with nintedanib/other drugs before 29 May 2020, ensured that the patients included in the study were treated after nintedanib became available in Japan as a treatment option for IPF, SSc-ILD, and PF-ILD, respectively.

### Variables and outcomes

Baseline characteristics, comorbidities (including major adverse cardiovascular events [MACE], gastro-esophageal reflux disease [GERD], obstructive sleep apnoea syndrome [OSAS], chronic obstructive pulmonary disease [COPD], lung cancer, and pulmonary hypertension as comorbidities of interest), clinical characteristics and laboratory results were recorded. If patients had multiple baseline measurements and data, the measurement before and nearest to the index date was used.

The number and classes of drugs for ILD treatment (i) prior to the initiation of antifibrotics, and (ii) concomitant with antifibrotics at the time of initiation of antifibrotic treatment were determined. The 18 classes of drugs examined were oral and IV glucocorticoids, oral and IV cyclophosphamide, azathioprine, oral ciclosporin, oral tacrolimus, mycophenolate or mycophenolate mofetil (MMF), mizoribine, oral and subcutaneous methotrexate (MTX), tumour necrosis factor (TNF) inhibitors, IL-5 inhibitors, IL-6 inhibitors, B-cell targeting therapies, T-cell targeting therapies, Janus kinase (JAK) inhibitors, and intravenous (IV) immunoglobulin (hereinafter referred to as ‘glucocorticoids/immunosuppressants for ILD treatment’), as well as N-acetylcysteine and the antifibrotics nintedanib and pirfenidone. Drugs for ILD treatment concomitant with antifibrotics were defined as those given within the 2 weeks before and after the index date for oral drugs and within the 2 months before and after the index date for injectable drugs. The number of drugs concomitant with antifibrotics were counted by class. For example, if a patient was prescribed glucocorticoids, oral tacrolimus, and JAK inhibitors concomitantly with nintedanib, the number of drugs for ILD treatment concomitant with nintedanib is three (classes). Drugs for ILD treatment prior to the initiation of antifibrotics were defined as those given after the first fibrosing ILD code and before the index date. The number of drugs prior to the initiation of antifibrotics were counted by class.

### Statistical analysis

Descriptive statistics were generated for all study variables. The summary statistics for continuous variables and the dose of each drug were the mean, standard deviation, minimum, 25th percentile (Q1), median, 75th percentile (Q3), and maximum. The drug dose was defined as the one during the prescription period including the index date or the average dose between the prescription periods before and after the index date. For categorical variables, the number of observations and the percentage were reported for each category. Subgroup analyses for the study outcomes were performed based on the dose of nintedanib at index (100 mg BID [200 mg/day], 150 mg BID [300 mg/day], and others), and the underlying disease (HP, exposure-related ILD [excluding HP], iNSIP, unclassifiable IIP, sarcoidosis, MCTD-ILD, PM/DM-ILD, RA-ILD, Sjögren ILD, SLE-ILD, SSc-ILD, other autoimmune ILD, and other ILDs), as defined by ICD-10 and disease codes (Supplementary Table S3). The study is descriptive in nature and no comparison or hypothesis tests were conducted.

A sensitivity analysis was conducted to determine any differences in the outcomes before and after the approval of nintedanib for PF-ILD on 29 May 2020, as prior to its approval, patients with PPF could be assigned IPF disease codes and prescribed nintedanib. IPF patients with the index date between 31 August 2015 and 28 May 2020 were analysed separately in the sensitivity analysis from those with the index date on or after 29 May 2020.

### Study design and oversight

The study was designed, conducted, and reported using best practice guidelines, with Institutional Review Board (IRB) approval from the Takahashi Clinic Ethics Committee on 5 December 2023 (IRB approval number: LNW00207). Hence, all objectives and methods were pre-specified and executed according to the study protocol.

## Results

### Patient disposition and baseline characteristics

The final patient cohorts are shown in Supplementary Figure S2. At the time of the index date, pirfenidone users in patients with IPF were the oldest in the study, with a mean (SD) age at 74.8 (7.3) years, with nintedanib users in patients with SSc-ILD the youngest on average at 65.6 (11.7) years (Table 1). The average age of patients with PPF tended to be lower in nintedanib users than in users of glucocorticoids/immunosuppressants (72.0 vs 74.1 years, respectively) (Table 1). The cohort with the highest proportion of patients over 80 years of age were patients with PPF treated with glucocorticoids/immunosuppressants (32.8%) (Table 1).

**Table 1:** Patient baseline characteristics

	Nintedanib users			Pirfenidone users	Glucocorticoids/immunosuppressants for ILD treatment	
	Patients with IPF	Patients with PPF	Patients with SSc-ILD	Patients with IPF	Patients with PPF	Patients with SSc-ILD
Total number of patients	657	4,160	676	418	18,403	698
Sex, n (%)						
Male	565 (86.0)	2,573 (61.9)	190 (28.1)	347 (83.0)	11,374 (61.8)	171 (24.5)
Female	92 (14.0)	1,587 (38.2)	486 (71.9)	71 (17.0)	7,029 (38.2)	527 (75.5)
Age at index date, years						
N	657	4,160	676	418	18,403	698
Mean (SD)	74.1 (7.4)	72.0 (9.2)	65.6 (11.7)	74.8 (7.3)	74.1 (10.9)	68.8 (12.1)
Median (IQR)	75 (70–79)	73 (68–78)	68 (58–73.3)	76 (70–80)	75 (69–81)	71 (62–77.8)
Min–Max	26–91	24–94	24–90	53–93	20–103	21–93
Age groups, years, n (%)						
18–39	1 (0.15)	27 (0.65)	18 (2.7)	–	214 (1.2)	13 (1.9)
40–49	2 (0.30)	95 (2.3)	52 (7.7)	–	438 (2.4)	47 (6.7)
50–59	14 (2.1)	266 (6.4)	117 (17.3)	16 (3.8)	1,061 (5.8)	86 (12.3)
60–69	136 (20.7)	873 (21.0)	185 (27.4)	76 (18.2)	2,979 (16.2)	167 (23.9)
70–79	349 (53.1)	2,151 (51.7)	245 (36.2)	218 (52.2)	7,677 (41.7)	254 (36.4)
≥ 80	155 (23.6)	748 (18.0)	59 (8.7)	108 (25.8)	6,034 (32.8)	131 (18.8)
Comorbidities, n (%)						
MACE	180 (27.4)	1,333 (32.0)	306 (45.3)	124 (29.7)	6,972 (37.9)	359 (51.4)
GERD	235 (35.8)	2,166 (52.1)	447 (66.1)	139 (33.3)	9,460 (51.4)	418 (59.9)
OSAS	7 (1.1)	66 (1.6)	5 (0.74)	7 (1.7)	267 (1.5)	7 (1.0)
COPD	265 (40.3)	1,566 (37.6)	223 (33.0)	182 (43.5)	5,154 (28.0)	139 (19.9)
Lung cancer	102 (15.5)	322 (7.7)	24 (3.6)	133 (31.8)	3,161 (17.2)	50 (7.2)
Pulmonary hypertension	17 (2.6)	234 (5.6)	171 (25.3)	8 (1.9)	378 (2.1)	141 (20.2)
Liver dysfunction	90 (13.7)	791 (19.0)	156 (23.1)	51 (12.2)	3,624 (19.7)	182 (26.1)
CKD	33 (5.0)	199 (4.8)	35 (5.2)	14 (3.4)	1,502 (8.2)	49 (7.0)
Diabetes	228 (34.7)	1,798 (43.2)	278 (41.1)	148 (35.4)	7,646 (41.6)	252 (36.1)
Hypertension	215 (32.7)	1,665 (40.0)	308 (45.6)	152 (36.4)	8,664 (47.1)	311 (44.6)
Hyperlipidaemia	148 (22.5)	1,322 (31.8)	227 (33.6)	116 (27.8)	5,592 (30.4)	223 (32.0)
CPI	22 (3.4)	190 (4.6)	40 (5.9)	12 (2.9)	827 (4.5)	33 (4.7)
TB	4 (0.61)	84 (2.0)	25 (3.7)	2 (0.48)	363 (2.0)	21 (3.0)
NTM	6 (0.91)	43 (1.0)	8 (1.2)	3 (0.72)	249 (1.4)	7 (1.0)
Pulmonary mycosis	12 (1.8)	68 (1.6)	9 (1.3)	7 (1.7)	266 (1.5)	10 (1.4)
Pulmonary *Aspergillus*	12 (1.8)	63 (1.5)	9 (1.3)	7 (1.7)	245 (1.3)	7 (1.0)
ILD clinical diagnosis, n (%)						
Exposure-related ILD (excluding HP)	–	50 (1.20)	–	–	332 (1.80)	–
HP	–	152 (3.7)	–	–	297 (1.6)	–
MCTD-ILD	–	59 (1.4)	–	–	117 (0.64)	–
Other autoimmune ILD	–	301 (7.2)	–	–	806 (4.4)	–
Other ILD	–	64 (1.5)	–	–	2,453 (13.3)	–
PM/DM-ILD	–	427 (10.3)	–	–	824 (4.5)	–
RA-ILD	–	114 (2.7)	–	–	291 (1.6)	–
SLE-ILD	–	223 (5.4)	–	–	492 (2.7)	–
SSc-ILD	–	524 (12.6)	676 (100)	–	599 (3.3)	698 (100)
Sarcoidosis	–	52 (1.3)	–	–	270 (1.5)	–
Sjögren ILD	–	213 (5.1)	–	–	678 (3.7)	–
Unclassifiable IIP	–	2,577 (62.0)	–	–	12,480 (67.8)	–
iNSIP	–	64 (1.5)	–	–	294 (1.6)	–
Unknown	–	–	–	–	63 (0.34)	–
Baseline drugs, n (%)						
Oral & IV glucocorticoids	154 (23.4)	1,960 (47.1)	375 (55.5)	93 (22.3)	17,287 (93.9)	564 (80.8)
Cyclophosphamide	4 (0.61)	92 (2.2)	42 (6.2)	0 (0)	126 (0.68)	25 (3.6)
Azathioprine	0 (0)	127 (3.1)	58 (8.6)	0 (0)	77 (0.42)	15 (2.2)
Oral ciclosporin	4 (0.61)	143 (3.4)	23 (3.4)	2 (0.48)	85 (0.46)	5 (0.72)
Oral tacrolimus	8 (1.2)	453 (10.9)	78 (11.5)	5 (1.2)	643 (3.5)	34 (4.9)
Mycophenolate	1 (0.15)	180 (4.3)	122 (18.1)	0 (0)	101 (0.55)	70 (10.0)
Mizoribine	0 (0)	16 (0.38)	9 (1.3)	0 (0)	34 (0.18)	2 (0.29)
Methotrexate	3 (0.46)	45 (1.1)	7 (1.0)	1 (0.24)	587 (3.2)	20 (2.9)
TNF inhibitors	2 (0.30)	27 (0.65)	2 (0.30)	1 (0.24)	77 (0.42)	5 (0.72)
IL-5 inhibitors	2 (0.30)	1 (0.02)	0 (0)	1 (0.24)	12 (0.07)	0 (0)
IL-6 inhibitors	3 (0.46)	64 (1.5)	29 (4.3)	1 (0.24)	77 (0.42)	12 (1.7)
B-cell targeting therapies	1 (0.15)	37 (0.89)	12 (1.8)	0 (0)	92 (0.50)	25 (3.6)
T-cell targeting therapies	1 (0.15)	55 (1.3)	10 (1.5)	1 (0.24)	59 (0.32)	3 (0.43)
JAK inhibitor	2 (0.30)	34 (0.82)	4 (0.59)	1 (0.24)	123 (0.67)	5 (0.72)
IV immunoglobulin	0 (0)	19 (0.46)	1 (0.15)	0 (0)	52 (0.28)	6 (0.86)
N-acetylcysteine	7 (1.1)	8 (0.19)	0 (0)	6 (1.4)	79 (0.43)	0 (0)
Pirfenidone	60 (9.1)	182 (4.4)	8 (1.2)	418 (100)	296 (1.6)	4 (0.57)
Nintedanib	657 (100)	4,160 (100)	676 (100)	43 (10.29)	637 (3.5)	76 (10.9)
Management, n (%)						
Oxygen therapy	86 (13.1)	727 (17.5)	85 (12.6)	32 (7.7)	777 (4.2)	28 (4.0)
Oxygen inhalation (palliative care)	147 (22.4)	951 (22.9)	92 (13.6)	91 (21.8)	7,692 (41.8)	178 (25.5)
Opioid use (palliative care)	20 (3.0)	120 (2.9)	16 (2.4)	25 (6.0)	1,202 (6.5)	34 (4.9)
Plasma exchange therapy	0 (0)	1 (0.02)	2 (0.30)	0 (0)	7 (0.04)	0 (0)
Endotoxin absorption therapy	1 (0.15)	2 (0.05)	0 (0)	0 (0)	3 (0.02)	0 (0)
Smoking experience						
N	175	857	66	110	6,226	108
Mean (SD)	966.6 (543.5)	809.1 (577.5)	608.7 (474.7)	942.6 (580.3)	878.8 (598.6)	679.0 (498.3)
Median (IQR)	900 (600–1,200)	740 (400–1,000)	520 (225–960)	820 (600–1,136.3)	800 (460–1,120)	600 (347.5–942.5)
Min–Max	20–2,880	4–5,000	14–1,800	20–3,540	1–6,000	16–2,640
Smoking quantity, cigarettes per day x years, n (%)						
> 0–200	10 (5.7)	93 (10.9)	16 (24.2)	7 (6.4)	614 (9.9)	20 (18.5)
> 200–400	17 (9.7)	127 (14.8)	13 (19.7)	9 (8.2)	780 (12.5)	19 (17.6)
> 400–600	22 (12.6)	156 (18.2)	13 (19.7)	15 (13.6)	969 (15.6)	22 (20.4)
> 600–800	24 (13.7)	148 (17.3)	4 (6.1)	24 (21.8)	946 (15.2)	14 (13.0)
> 800–1,000	40 (22.9)	125 (14.6)	7 (10.6)	21 (19.1)	1,057 (17.0)	15 (13.9)
> 1,000	62 (35.4)	208 (24.3)	13 (19.7)	34 (30.9)	1,860 (29.9)	18 (16.7)
Height, cm						
N	274	1,694	214	170	13,198	396
Mean (SD)	162.4 (8.5)	159.9 (9.1)	157.3 (8.3)	160.9 (8.3)	159.7 (9.3)	155.9 (8.7)
Median (IQR)	163 (158–168)	161 (153–166)	157 (151.3–163)	162 (156.3–166)	160 (153–166)	155 (150–162)
Min–Max	130–181	131–187	138–179	141–180	69–190	132–186
Body weight, kg						
N	276	1,698	214	169	13,362	397
Mean (SD)	62.1 (12.5)	60.5 (12.7)	57.9 (12.2)	61.1 (12.3)	58.2 (13.5)	52.6 (11.4)
Median (IQR)	61.8 (54.0–70.5)	60.1 (51.6–68.4)	56.8 (48.5–63.9)	62.1 (51.7–70)	57.5 (49.7–65.3)	51.4 (44.8–59.8)
Min–Max	33.4–108.8	26.3–120.7	29.9–96.7	28.0–94.5	24.8–514.0	28.0–97.0
BMI, kg/m^2^						
N	274	1,692	214	169	13,165	396
Mean (SD)	23.4 (3.7)	23.6 (4.0)	23.4 (4.4)	23.5 (3.9)	22.7 (5.2)	21.5 (3.7)
Median (IQR)	23.5 (20.8–25.9)	23.5 (21.0–26.0)	23.2 (20.4–26.0)	23.6 (20.9–25.7)	22.5 (20.1–24.9)	21.3 (18.8–23.9)
Min–Max	15.2–33.6	12.9–40.2	13.3–40.2	12.6–36.0	10.9–344.9	12.3–33.5
KL-6, U/mL						
N	59	466	43	67	1,597	51
Mean (SD)	1,179.9 (951.2)	1,235.0 (1,003.7)	1,165.8 (792.8)	946.5 (860.7)	1,029.4 (1,306.4)	948.3 (1,091.2)
Median (IQR)	827 (582.5–1,311)	928.5 (613–1,540.3)	939 (683.5–1,352)	673 (447.5–1,133)	651 (360–1,211)	641 (362–1,022.5)
Min–Max	306–4,731	182–9,249	231–3,819	223–5,407	78–15,900	165–6,578
LDH, U/L						
N	72	476	49	75	2,026	67
Mean (SD)	236.7 (71.5)	234.8 (58.8)	225.0 (50.4)	226.1 (89.1)	265.6 (189.7)	243.1 (123.3)
Median (IQR)	228.5 (186.8–270)	226 (193–261)	222 (190–257)	203 (171.5–239)	229 (187–295.8)	212 (185.5–261.5)
Min–Max	125–597	107–455	146–353	121–672	78–5,115	136–998
CRP, mg/dL						
N	72	482	49	78	2,118	70
Mean (SD)	1.1 (2.6)	0.78 (2.3)	0.55 (1.2)	1.4 (2.8)	4.6 (6.4)	2.6 (4.8)
Median (IQR)	0.22 (0.11–0.92)	0.20 (0.08–0.58)	0.10 (0.04–0.56)	0.30 (0.14–1.2)	1.5 (0.25–7.3)	0.38 (0.10–2.2)
Min–Max	0.03–17.0	0.01–28.7	0.01–7.4	0.02–17.8	0.01–44.3	0.01–22.9
Neutrophil count, %						
N	53	376	37	44	1,662	56
Mean (SD)	66.7 (12.2)	66.7 (12.1)	66.8 (12.4)	68.2 (11.3)	69.9 (12.1)	68.0 (11.2)
Median (IQR)	66.2 (58.9–74.3)	67.1 (58.1–75.0)	66.2 (58.2–76.4)	68.7 (59.7–76.2)	70.6 (61.7–78.7)	67.9 (60.5–76.0)
Min–Max	28.3–89.1	29.9–94.3	35–88.1	47.1–89.8	15.5–98.1	41.6–95.7
WBC count, cells/μL						
N	75	501	49	84	2,152	69
Mean (SD)	8,158.4 (2,825.3)	8,140.8 (2,917.2)	7,717.8 (2,653.3)	8,776.8 (3,499.5)	8,268.8 (3,576.2)	8,227.4 (4,080.6)
Median (IQR)	7,900 (6,100–9,100)	7,700 (6,200–9,340)	7,400 (5,800–9,000)	8,040 (6,772.5–9,600)	7,500 (5,967.5–9,782.5)	7,400 (5,490–9,470)
Min–Max	4,100–21,100	2,600–29,900	3,390–14,400	4,230–25,800	96–45,040	3,500–23,580
Monocyte count, %						
N	52	412	40	27	1,735	57
Mean (SD)	6.8 (4.4)	6.0 (2.1)	6.6 (2.2)	5.9 (2.5)	6.5 (3.0)	6.9 (2.8)
Median (IQR)	6 (5.2–7.0)	5.9 (4.7–7.2)	6.5 (4.9–7.8)	5.6 (4.5–6.8)	6.1 (4.9–7.8)	6.6 (5.3–8.2)
Min–Max	1.3–33.5	1.0–14.0	1.8–10.9	1.5–13.6	0.40–36.6	1.2–18.0
Lymphocyte count, %						
N	52	411	40	27	1,720	58
Mean (SD)	23.3 (10.2)	24.1 (10.6)	22.8 (10.8)	22.4 (10.6)	19.8 (10.2)	20.7 (10.0)
Median (IQR)	21.3 (16.6–30.9)	22.8 (16.5–32.0)	23.4 (13.8–30.2)	21.8 (13.9–30.4)	18.6 (12–26.1)	20.1 (12.9–28.3)
Min–Max	7.0–50.0	4.0–56.3	4.8–48.9	5.9–41.6	0.90–85.0	2.5–50.2
Neutrophil to lymphocyte ratio						
N	47	357	35	25	1,501	50
Mean (SD)	3.9 (2.7)	3.9 (3.2)	4.3 (3.5)	4.5 (3.9)	5.6 (6.4)	5.0 (6.0)
Median (IQR)	3.3 (1.9–4.5)	3 (1.9–4.5)	2.9 (1.9–5.9)	2.9 (2–4.9)	3.7 (2.4–6.5)	3.5 (2.1–4.8)
Min–Max	0.57–12.0	0.53–22.5	0.72–18.4	1.2–15.2	0.39–109.0	0.83–31.9

*BMI* Body mass index, *CKD* Chronic kidney disease, *COPD* Chronic obstructive pulmonary disease, *CPI* Chronic pulmonary infection, *CRP* C-reactive protein, *GERD* Gastroesophageal reflux disease, *HP* Hypersensitivity pneumonitis, *IIP* Idiopathic interstitial pneumonia, *IL* Interleukin, *ILD* Interstitial lung disease, *iNSIP* Idiopathic non-specific interstitial pneumonia, *IPF* Idiopathic pulmonary fibrosis, *IQR* Interquartile range, *IV* Intravenous, *JAK* Janus kinase, *KL-6* Krebs von den Lungen-6, *LDH* Lactate dehydrogenase, *MACE* Major adverse cardiovascular event, *max* maximum, *MCTD* Mixed connective tissue disease, *min* minimum, *NTM* Non-tuberculosis mycobacteria, *OSAS* Obstructive sleep apnoea syndrome, *PM/DM* Polymyositis/dermatomyositis, *PPF* Progressive pulmonary fibrosis, *RA* Rheumatoid arthritis, *SD* Standard deviation, *SLE* Systemic lupus erythematosus, *SSc* Systemic sclerosis, *TB* Tuberculosis, *TNF* Tumour necrosis factor, *WBC* White blood cell

COPD was the most common comorbidity of interest in patients with IPF, affecting over 40% of the population, while GERD had the highest prevalence in patients with PPF and SSc-ILD, affecting over 50% of the population (Table 1), and up to 70% of nintedanib users in patients with PM/DM-ILD and SLE-ILD (Supplementary Table S4). The top ILD diagnoses in nintedanib users in patients with PPF were unclassifiable IIP (62.0%), SSc-ILD (12.6%), PM/DM-ILD (10.3%), and other autoimmune ILDs (7.2%) while for users of glucocorticoids/immunosuppressants for ILD treatment in patients with PPF they were unclassifiable IIP (67.8%), other ILDs (13.3%), PM/DM-ILD (4.5%), and other autoimmune ILDs (4.4%) (Table 1). Among 657 nintedanib users and 418 pirfenidone users in patients with IPF, 60 (9.1%) and 43 (10.3%) were prescribed pirfenidone and nintedanib during the 6 months up to the index date (i.e., the look-back period), respectively. The average age of patients with non-CTD-ILDs (HP, exposure-related ILD [excluding HP], iNSIP, sarcoidosis, and unclassifiable IIP) tended to be older than those with CTD-ILDs (RA-ILD, SSc-ILD, MCTD-ILD, SLE-ILD, PM/DM-ILD, and Sjögren ILD; Supplementary Table S4). The average age of patients receiving 200 mg/day of nintedanib tended to be older than those receiving 300 mg/day, and the average body weight and body mass index (BMI) of those receiving 200 mg/day of nintedanib tended to be lower than those receiving 300 mg/day (Supplementary Tables S5-S6).

### Concomitant ILD treatment at initiation of antifibrotics

**Fig. 1:**
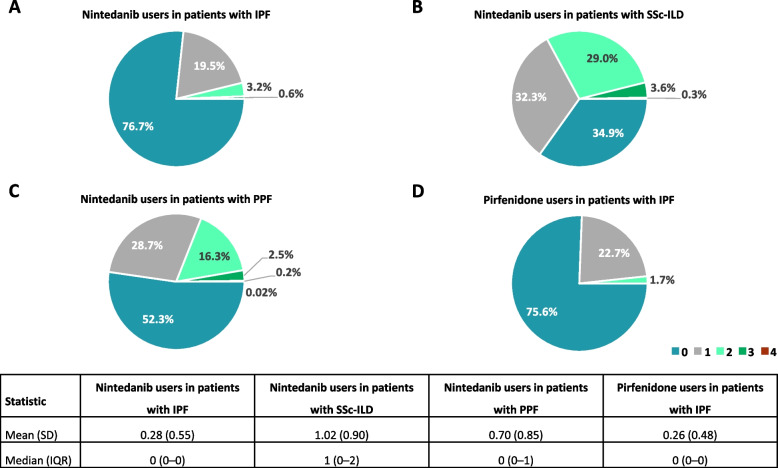
Number of drugs for ILD treatment concomitant with antifibrotics at the time of initiation of antifibrotic treatment. ILD, interstitial lung disease; IPF, idiopathic pulmonary fibrosis; IQR, interquartile range; PPF, progressive pulmonary fibrosis; SD, standard deviation; SSc, systemic sclerosis

**Fig. 2:**
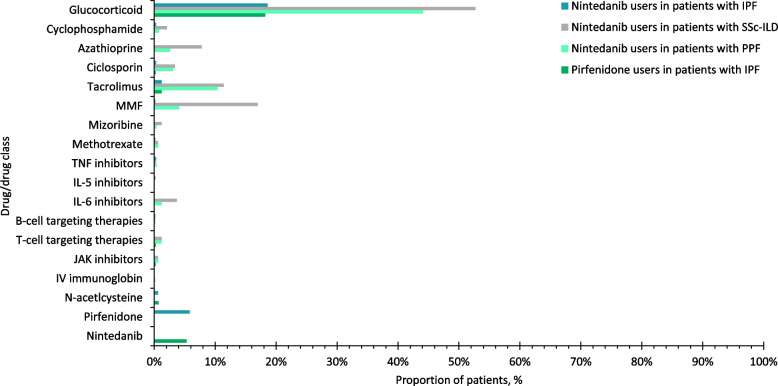
Percentage of patients concomitantly treated with each drug (by class). IL, interleukin; IPF, idiopathic pulmonary fibrosis; IV, intravenous; JAK, Janus kinase; MMF, mycophenolate mofetil; PPF, progressive pulmonary fibrosis; SSc-ILD, systemic sclerosis-interstitial lung disease; TNF, tumour necrosis factor

**Fig. 3:**
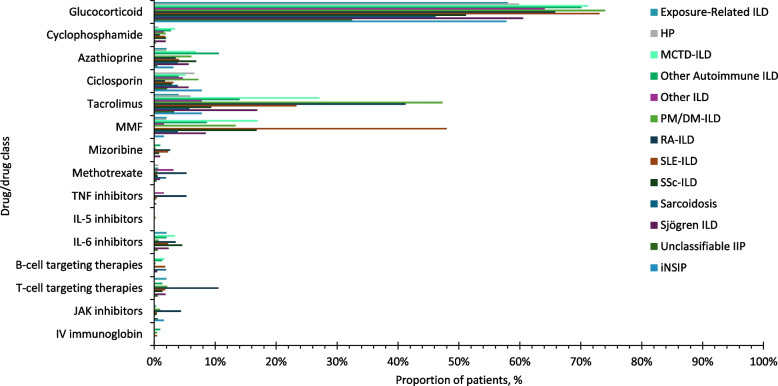
Percentage of nintedanib users in patients with PPF concomitantly treated with each drug (by class) by underlying disease. HP, hypersensitivity pneumonitis; IIP, idiopathic interstitial pneumonia; IL, interleukin; ILD, interstitial lung disease; iNSIP, idiopathic non-specific interstitial pneumonia; JAK, Janus kinase; MCTD, mixed connective tissue disease; MMF, mycophenolate mofetil; PM/DM, polymyositis/dermatomyositis; PPF, progressive pulmonary fibrosis; RA, rheumatoid arthritis; SLE, systemic lupus erythematosus; SSc, systemic sclerosis; TNF, tumour necrosis factor

Over three quarters of patients with IPF were prescribed nintedanib or pirfenidone as monotherapy for ILD treatment (Figs. 1A; D). The most common concomitant drug class was glucocorticoids, which were prescribed in 18.6% of nintedanib users and 18.2% of pirfenidone users, respectively, in patients with IPF (Fig. 2). In patients with IPF, among the 657 nintedanib users, 38 (5.8%) were prescribed pirfenidone, and among the 418 pirfenidone users, 22 (5.3%) were prescribed nintedanib within 2 weeks of the index date. Approximately two thirds of patients with SSc-ILD were prescribed nintedanib concomitantly with one or more glucocorticoids/immunosuppressants for ILD treatment (Fig. 1B). The most common drugs prescribed alongside nintedanib in patients with SSc-ILD were glucocorticoids (52.7%) and mycophenolate (17.0%) (Fig. 2). Around half of patients with PPF were prescribed nintedanib along with one or more glucocorticoids/immunosuppressants for ILD treatment (Fig. 1C). Concomitant glucocorticoid use was 44.1% for nintedanib users with PPF. Among immunosuppressants, the most common concurrent drug was tacrolimus, followed by mycophenolate and ciclosporin in patients with PPF (Fig. 2). Among patients with PPF, over two thirds of nintedanib users in patients with RA-ILD, PM/DM-ILD, MCTD-ILD, SLE-ILD, Sjögren-ILD, other autoimmune ILDs and other ILDs were prescribed nintedanib in combination with one or more glucocorticoids/immunosuppressants for ILD treatment at the time of initiation of antifibrotics (Supplementary Figure S3). Glucocorticoids was the most common concomitant drug class in these subgroups, followed by tacrolimus and mycophenolate (Fig. 3). The doses of drugs used in combination with antifibrotics are shown in Supplementary Tables S7 and S8.

### ILD treatment prior to the initiation of antifibrotics

**Fig. 4:**
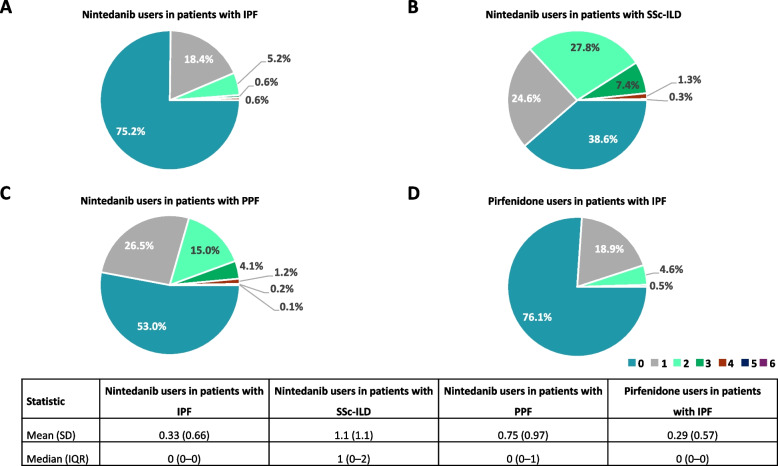
Number of drugs for ILD treatment prior to the initiation of antifibrotics. ILD, interstitial lung disease; IPF, idiopathic pulmonary fibrosis; IQR, interquartile range; PPF, progressive pulmonary fibrosis; SD, standard deviation; SSc, systemic sclerosis

**Fig. 5:**
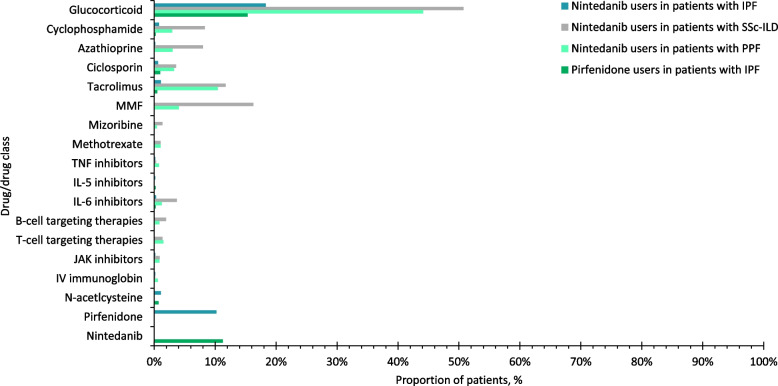
Percentage of patients treated with each drug or class prior to the initiation of antifibrotics (by class). IL, interleukin; IPF, idiopathic pulmonary fibrosis; IV, intravenous; JAK, Janus kinase; MMF, mycophenolate mofetil; PPF, progressive pulmonary fibrosis; SSc-ILD, systemic sclerosis-interstitial lung disease; TNF, tumour necrosis factor

**Fig. 6:**
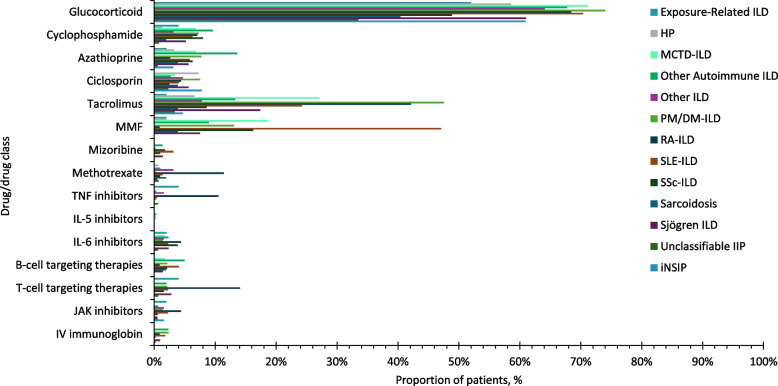
Percentage of nintedanib users in patients with PPF treated with each drug or class prior to the initiation of antifibrotics (by class) by underlying disease. HP, hypersensitivity pneumonitis; IIP, idiopathic interstitial pneumonia; IL, interleukin; ILD, interstitial lung disease; iNSIP, idiopathic non-specific interstitial pneumonia; JAK, Janus kinase; MCTD, mixed connective tissue disease; MMF, mycophenolate mofetil; PM/DM, polymyositis/dermatomyositis; PPF, progressive pulmonary fibrosis; RA, rheumatoid arthritis; SLE, systemic lupus erythematosus; SSc, systemic sclerosis; TNF, tumour necrosis factor

Over three quarters of patients with IPF were prescribed nintedanib or pirfenidone as first-line therapy (Fig. 4A; D). The most common drug class prescribed prior to the initiation of antifibrotics was glucocorticoids (Fig. 5). In patients with IPF, among the 657 nintedanib users, 67 (10.2%) were prescribed pirfenidone, and among the 418 pirfenidone users 47 (11.2%) were prescribed nintedanib, prior to the index date. In patients with SSc-ILD 61.4% were prescribed nintedanib after one or more glucocorticoids/immunosuppressants for ILD treatment (Fig. 4B). The most common drug for ILD treatment prior to the initiation of antifibrotics in patients with SSc-ILD was glucocorticoids, followed by mycophenolate and tacrolimus (Fig. 5). Around half of patients with PPF were prescribed nintedanib after one or more glucocorticoids/immunosuppressants for ILD treatment (Fig. 4C). Among immunosuppressants, the most common drug for ILD treatment prior to the initiation of antifibrotics in patients with PPF was tacrolimus, followed by mycophenolate and ciclosporin (Fig. 5). Among patients with PPF, over three quarters of patients with RA-ILD, MCTD-ILD, PM/DM-ILD, and SLE-ILD were prescribed nintedanib after one or more glucocorticoids/immunosuppressants prior to the initiation of antifibrotics (Supplementary Figure S4). The most common drug prescribed prior to the initiation of antifibrotics was glucocorticoids in these patients, followed by tacrolimus and mycophenolate (Fig. 6).

### Sensitivity analysis

The results of the sensitivity analysis conducted to determine any differences in outcomes in patients with IPF before and after the approval of nintedanib for PF-ILD were consistent with the main analysis.

## Discussion

To the best of our knowledge, this is the first observational, non-interventional study of patients with IPF, SSc-ILD, and PPF to examine characteristics and treatment patterns of antifibrotics in real-world clinical practice, using a major health insurance claims database. In patients with IPF, the predominant use of antifibrotics as monotherapy and first-line therapy aligns with clinical guidelines and clinical trial data. The frequent use of antifibrotic drugs after, and in combination with, glucocorticoids and other immunosuppressants in patients with SSc-ILD and PPF, especially in patients with autoimmune-related PPF, suggests that antifibrotic drugs are used for cases where disease progression is observed despite treatment with anti-inflammatory therapy. While the efficacy of antifibrotics in slowing lung function decline has been demonstrated in clinical trials, our data underscore important differences in their usage patterns across various disease populations in real-world clinical practice.

In this study patient characteristics in general were similar across patients with IPF, SSc-ILD, and PPF, although patients with autoimmune ILD were younger and more likely to be women than patients with IPF and non-autoimmune ILD, which is consistent with previous reports [28, 29]. The average age of 200 mg/day nintedanib users tended to be older, with lower average body weight and BMI, than 300 mg/day users. This may be because in clinical practice physicians took patients’ characteristics, such as older age, and lower body weight and BMI, into account when prescribing a lower dose of nintedanib, although this is not beyond speculation, as clinical characteristics are obtained from only a certain percentage of patients in this study. Previous INPULSIS subgroup analysis by age showed that the subgroup aged ≥ 75 years had a lower mean weight BMI and that the proportion of patients with adverse events leading to treatment discontinuation was greater in patients aged ≥ 75 years than < 75 years in both the nintedanib and placebo groups [30]. In addition, a multivariate analysis in an interim report of a post-marketing surveillance study of nintedanib in Japan reported that patients aged > 75 years or with body surface area of < 1.58 m^2^ at baseline were more likely to discontinue nintedanib treatment [31]. Therefore, there may be an unmet need for drugs for the treatment of fibrotic ILDs with more acceptable tolerability in elderly patients and/or patients with low BMI in clinical practice. Several drug candidates are in clinical development for fibrotic ILD that have novel modes of action and improved safety and tolerability profiles in the early stages of development [32]. The most advanced in development among them is nerandomilast (BI 1015550), which is an oral preferential phosphodiesterase 4B (PDE4B) inhibitor with approximately ninefold selectivity for inhibition of PDE4B compared with PDE4D [33]. In a phase II trial of patients with IPF, nerandomilast prevented a decline in forced vital capacity over 12 weeks and had an acceptable safety profile both as a monotherapy and in addition to background antifibrotic treatment [34]. Nerandomilast is being investigated in two phase III trials: FIBRONEER-IPF™ in patients with IPF and FIBRONEER-ILD™ in patients with PPF [35, 36]. Topline data from FIBRONEER-IPF™ and FIBRONEER-ILD™ show that nerandomilast is effective in patients with IPF and PPF with both trials meeting their primary endpoint, which was the absolute change from baseline in forced vital capacity (mL) at week 52 versus placebo [37, 38].

In patients with IPF most (approximately three quarters) were prescribed antifibrotics as monotherapy for ILD treatment and as first-line therapy. An Official ATS/ERS/JRS/ALAT Clinical Practice Guideline: Treatment of Idiopathic Pulmonary Fibrosis conditionally recommended clinical use of pirfenidone and nintedanib for the treatment of IPF in 2015 [21]. The Japanese Respiratory Society (JRS) 2017 guideline for the treatment of IPF weakly recommended pirfenidone and nintedanib for the treatment of IPF [39]. These international and Japanese guidelines strongly recommend that patients with IPF should not be treated with glucocorticoid monotherapy for IPF treatment or glucocorticoid and immunomodulator combination therapy [39, 40]. Therefore, the large proportion of patients with IPF receiving antifibrotic treatment as monotherapy for ILD treatment and as first-line treatment is expected. The JRS 2017 guideline for the treatment of IPF did not make a conclusion on pirfenidone and nintedanib combination therapy, however, a 2023 update to the guideline recommended against pirfenidone and nintedanib combination therapy [26]. This could be a contributing explanation for the relatively low proportion of patients with IPF treated concomitantly with pirfenidone and nintedanib.

In patients with SSc-ILD the use of other glucocorticoid/immunosuppressant drugs prior to, and concomitantly with, the use of nintedanib was common. In these patients just 34.9% were prescribed nintedanib as monotherapy for ILD treatment, and 38.6% as first-line therapy. The 2020 guide for the diagnosis and treatment of CTD-ILD suggested treatment of SSc-ILD with 1) oral cyclophosphamide (or IV cyclophosphamide may be considered), followed by azathioprine or MMF, 2) MMF, 3) nintedanib, or 4) tocilizumab as choices for first-line treatment [41]. In the SENSCIS trial, prior use, and concomitant use of glucocorticoids, MMF or MTX was permitted. In fact, 68.9%, 48.4%, and 6.6% of the enrolled patients received glucocorticoids, MMF and MTX, respectively, at baseline [8, 42]. Therefore, the greater concomitant use of other drugs with nintedanib, and greater use prior to nintedanib treatment in patients with SSc-ILD compared with patients with IPF is expected.

In patients with PPF, approximately half of patients were prescribed nintedanib concomitantly with other glucocorticoids/immunosuppressant drugs. Furthermore, approximately half of patients with PPF were prescribed nintedanib after one or more glucocorticoids/immunosuppressant drugs. Those with non–autoimmune-related PPF were more likely to receive nintedanib as monotherapy for ILD treatment (32.8%–65.8%) and first-line therapy (34.4%–65.1%) than those with autoimmune-related PPF (11.2%–37.8% and 16.6%–41.8%, respectively). The INBUILD trial showed the efficacy of nintedanib in patients with PPF whose fibrosing ILD progressed despite management considered appropriate in clinical practice [5]. In addition, ATS/ERS/JRS/ALAT guidelines conditionally recommend nintedanib treatment for patients with PPF who have failed standard management for fibrotic ILD including immunosuppressive treatments [4]. Therefore, the greater concomitant use of other drugs with nintedanib, and greater use prior to nintedanib treatment in patients with PPF compared with patients with IPF, is expected.

In patients with PPF the use of other glucocorticoids/immunosuppressant drugs prior to the use of nintedanib and concomitantly with nintedanib varied across underlying disease type. The 2022 JRS guidelines for HP suggested nintedanib as a treatment option in patients with moderate cases or in those whose disease progresses despite antigen avoidance [43]. In addition, 2022 JRS guidelines for IIP suggested nintedanib as a treatment option in patients with fibrotic iNSIP [25]. Therefore, the lower use of other drugs for ILD treatment prior to, and concomitantly with nintedanib in patients with non–autoimmune fibrotic ILD, including HP and iNSIP, compared to those with autoimmune fibrotic ILD, may be explained by differing guidelines and treatment options for the underlying cause of PPF. Overall, antifibrotic use in patients with PPF in this study is in line with the following existing literature. Interim analysis of a 2-year post-marketing surveillance study of nintedanib in patients with PPF in Japan reported 50.1% and 20.8% of patients were receiving glucocorticoids and tacrolimus concomitantly with nintedanib, respectively [44]. This is in-line with the use of these drugs reported here. In a real-world observational study of patients with PPF treated with nintedanib in the United Kingdom, 90% were receiving concurrent immunosuppressive drugs, most commonly prednisolone and MMF. Furthermore, 60% of patients had unchanged immunosuppressive treatment following initiation of nintedanib, and a further 23% increased their immunosuppressive treatment [45]. This is consistent with the frequent use of glucocorticoid/immunosuppressive drugs used concomitantly with nintedanib in patients with PPF in this study. Additional studies in real-world cohorts of patients with PF-ILD in the United Kingdom, and RA-ILD in Massachusetts, United States, also report frequent concomitant use of immunomodulatory and immunosuppressive drugs alongside antifibrotic use, suggesting that this is consistent across a range of healthcare systems globally [46, 47].

Although international IPF, SSc-ILD and PPF treatment guidelines provide treatment options for ILDs, there remain clinical questions about the timing of prescription of antifibrotics in the context of available options to treat ILDs. Current PPF treatment guidelines do not give clarity as to whether to continue immunomodulatory medications for patients with ILDs when the progressive PPF occurs, at what point to introduce an antifibrotic, or whether combination therapy is recommended [48]. In this study, we show that currently in real-world practice combination therapy is common in patients with SSc-ILD and PPF while antifibrotic monotherapy is common in patients with IPF. Therefore, the information presented in this study is of valuable importance to physicians who prescribe antifibrotics globally and highlights the need for further clinical trials to investigate the benefits of combination immunomodulatory and antifibrotic therapy in SSc-ILD and PPF, and to inform international treatment guidelines. However, this study has several limitations. Firstly, as the disease definition has not been validated there is the possibility of misidentification of ILD based on ICD codes and misclassification of underlying diseases. Secondly, oral and IV glucocorticoids/immunosuppressants/DMARDs for ILD might include those for the treatment of the underlying disease or CTD rather than the ILD. Thirdly, although IV glucocorticoids were investigated, it is possible glucocorticoids administered through other routes were included. For example, IV glucocorticoids might have included intramuscular and intra-articular injection although they cannot be distinguishable from IV injection using only drug codes. Another limitation is that laboratory test results were only available for a small percentage of patients. Furthermore, data on smoking experience and clinical characteristics (height, body weight, and BMI) were only available for a limited number of patients who were hospitalized. Height, body weight, and BMI could affect the prescription dose of nintedanib and a lack of complete data for these characteristics restricted further analysis by subgroups to confirm this. Additionally, as the MDV database lacks pulmonary function test results, it was not possible to collect data on and perform subgroup analysis by, the severity of ILD, which could affect the choice and number of drugs prescribed prior to antifibrotics and the number of concomitant drugs prescribed. Finally, patients in Japan have free access to healthcare facilities, and data from other hospitals not part of the MDV database were not included in this study.

## Conclusions

These results provide real-world evidence on the use of antifibrotics in clinical practice. Most patients with IPF were prescribed antifibrotics as monotherapy for ILD treatment whereas antifibrotics were used concomitantly with glucocorticoids and immunosuppressants in many patients with SSc-ILD, autoimmune-related PPF, and non–autoimmune-related PPF. Most patients with IPF were prescribed antifibrotics as first-line therapy. Patients with SSc-ILD, autoimmune-related PPF, and non–autoimmune-related PPF were more likely to be prescribed nintedanib after glucocorticoids/immunosuppressants than patients with IPF. Since there are no significant differences between countries in treatment recommendations for IPF, SSc-ILD, and PPF, the results of this study are of relevance globally.

## Supplementary Information

Supplementary Material 1

Supplementary Material 2

## Data Availability

The claims data that support the findings of this study are available from Medical Data Vision Co., Limited, but restrictions apply to the availability of these data, which were used under license for the current study, and so are not publicly available. Data are however available from Tomohiro Ito (tomohiro.ito@boehringer-ingelheim.com) and Haikun Bao (haikun.bao@boehringer-ingelheim.com) upon reasonable request and with permission from Medical Data Vision Co., Limited.

## Abbreviations

ACR: American College of Rheumatology
ALAT: Latin American Thoracic Association
ATS: American Thoracic Society
BID: Twice a day
BMI: Body mass index
CHEST: American College of Chest Physicians
COPD: Chronic obstructive pulmonary disease
CRP: C-reactive protein
CTD: Connective tissue disease
DMARDs: Disease-modifying anti-rheumatic drugs
ERS: European Respiratory Society
GERD: Gastroesophageal reflux disease
HP: Hypersensitivity pneumonitis
IIP: Idiopathic interstitial pneumonia
IL: Interleukin
ILD: Interstitial lung disease
iNSIP: Idiopathic non-specific interstitial pneumonia
IPF: Idiopathic pulmonary fibrosis
IQR: Interquartile range
IV: Intravenous
JAK: Janus kinase
JRS: Japanese Respiratory Society
KL-6: Krebs von den Lungen-6
LDH: Lactate dehydrogenase
MACE: Major adverse cardiovascular event
MCTD: Mixed connective tissue disease
MDV: Medical Data Vision
MMF: Mycophenolate mofetil
OSAS: Obstructive sleep apnoea syndrome
PDE: Phosphodiesterase
PF-ILD: Progressive fibrosing-interstitial lung disease
PM/DM: Polymyositis/dermatomyositis
PPF: Progressive pulmonary fibrosis
Q: Quartile
RA: Rheumatoid arthritis
SARD: Systemic autoimmune rheumatic disease
SD: Standard deviation
SLE: Systemic lupus erythematosus
SSc: Systemic sclerosis
TNF: Tumour necrosis factor
WBC: White blood cell
