# Supplementary material for: Patient characteristics and pharmacologic treatment patterns in antifibrotic-treated patients with fibrosing interstitial lung diseases: real-world results from a claims database
Source: BMC Pulm Med. 2025 May 22;25:253. doi: 10.1186/s12890-025-03713-x (PMC12096679; doi:10.1186/s12890-025-03713-x)
Supplement: Supplementary file 2 — Supplementary Material 2 [file 12890_2025_3713_MOESM2_ESM.pdf]

**Supplementary Figure S1.** Schematic of the study design for (**A**) patients with IPF; (**B**) patients with SSc-ILD; and (**C**) patients with PPF.

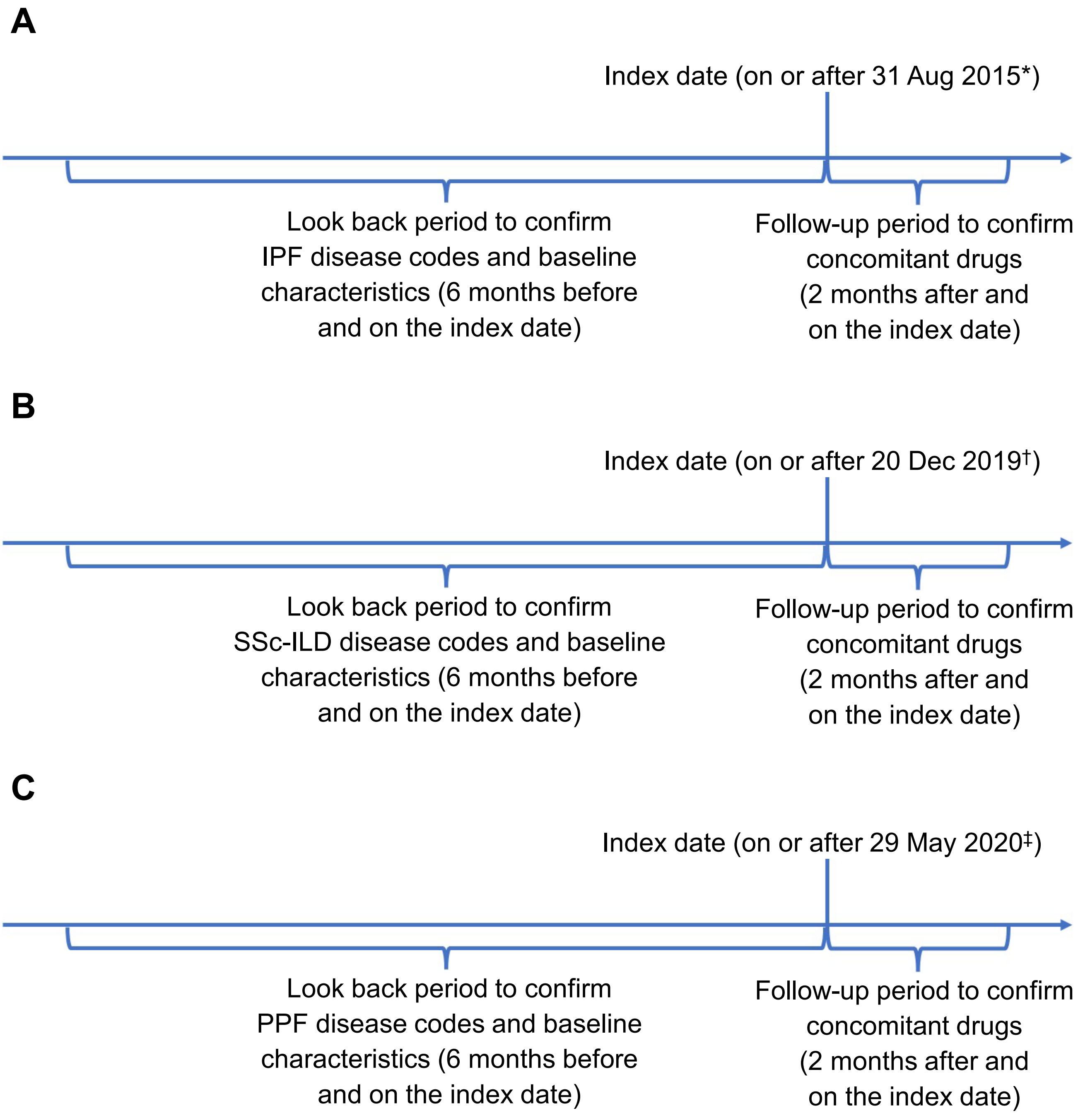


*The date nintedanib became commercially available. †The date nintedanib was approved for SSc-ILD. ‡The date nintedanib was approved for PF-ILD.

CTD, connective tissue disease; IPF, idiopathic pulmonary fibrosis; PF-ILD, progressive fibrosing-interstitial lung disease; PPF, progressive pulmonary fibrosis; SSc-ILD; systemic sclerosis-interstitial lung disease.

**Supplementary Figure S2.** Patient flow diagram for (**A**) nintedanib users in patients with IPF; (**B**) pirfenidone users in patients with IPF; (**C**) nintedanib users in patients with PPF; (**D**) users of oral and IV glucocorticoids/immunosuppressive drugs/DMARDs for ILD treatment in patients with PPF; (**E**) nintedanib users in patients with SSc-ILD; and (**F**) users of oral and IV glucocorticoids/immunosuppressive drugs/DMARDs for ILD treatment in patients with SSc-ILD.

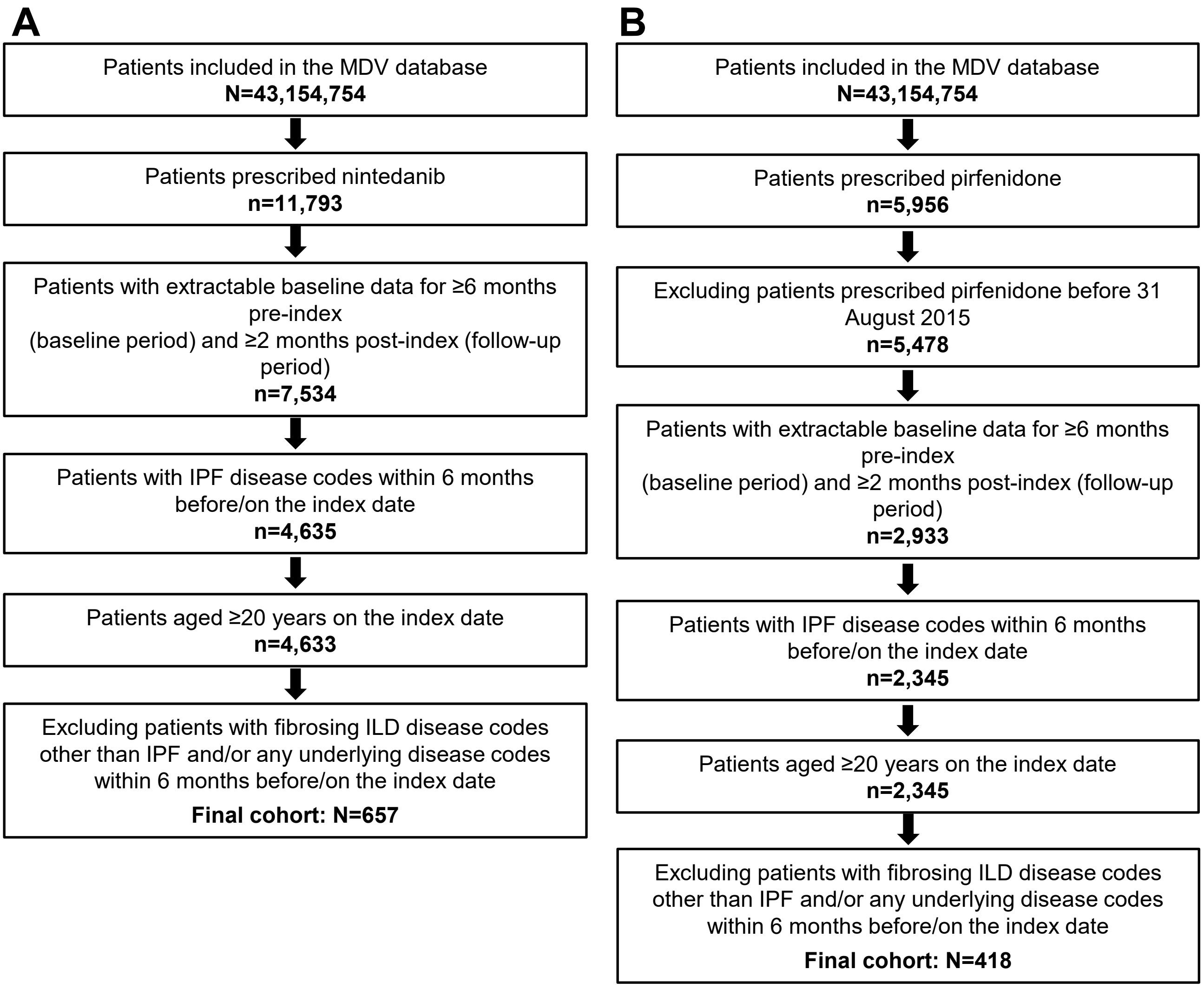

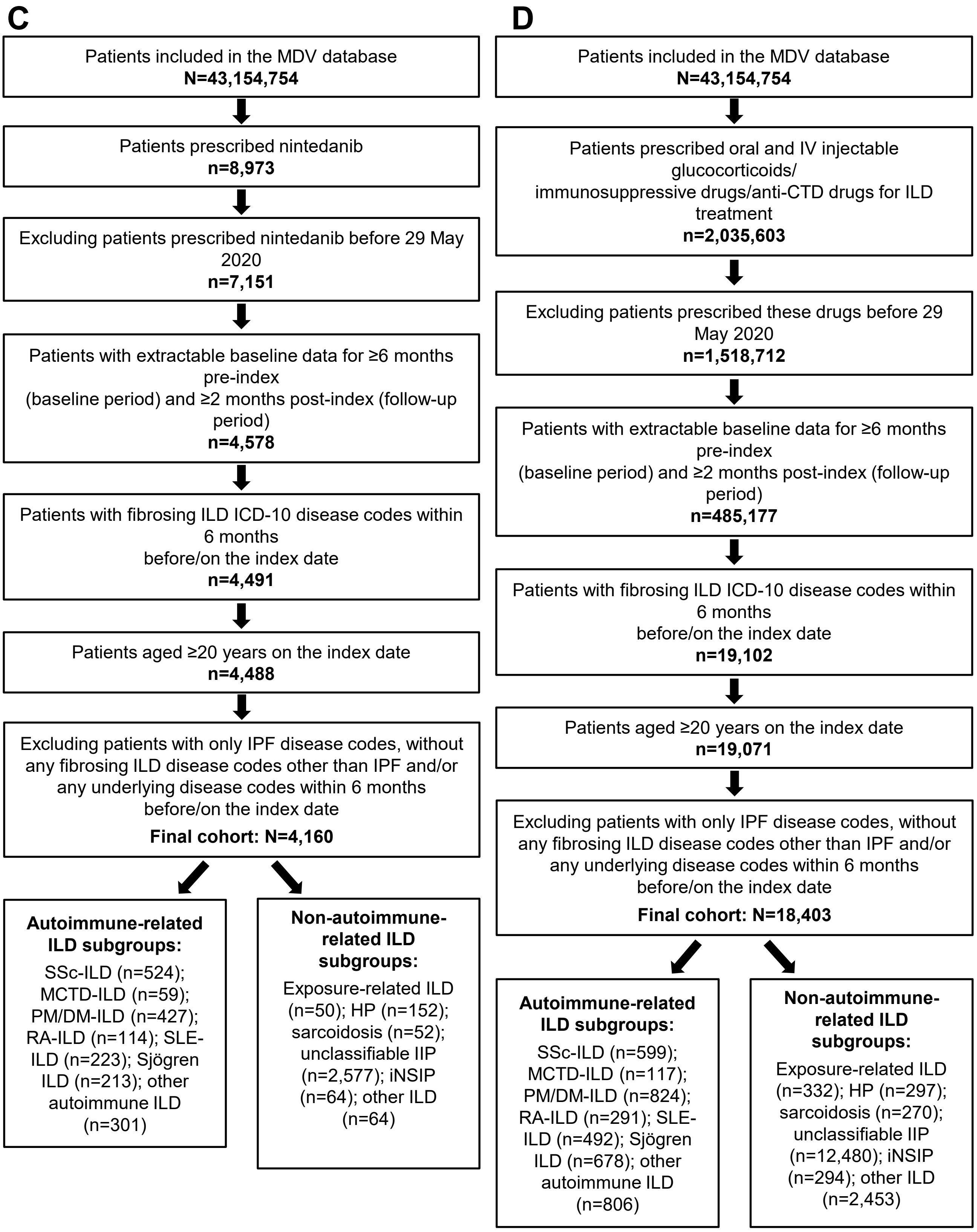
C
Patients included in the MDV database
N=43,154,754
Patients prescribed nintedanib
n=8,973
Excluding patients prescribed nintedanib before 29 May 2020
n=7,151
Patients with extractable baseline data for ≥6 months pre-index (baseline period) and ≥2 months post-index (follow-up period)
n=4,578
Patients with fibrosing ILD ICD-10 disease codes within 6 months before/on the index date
n=4,491
Patients aged ≥20 years on the index date
n=4,488
Excluding patients with only IPF disease codes, without any fibrosing ILD disease codes other than IPF and/or any underlying disease codes within 6 months before/on the index date
Final cohort: N=4,160
Autoimmune-related ILD subgroups:
SSc-ILD (n=524); MCTD-ILD (n=59); PM/DM-ILD (n=427); RA-ILD (n=114); SLE-ILD (n=223); Sjögren ILD (n=213); other autoimmune ILD (n=301)
Non-autoimmune-related ILD subgroups:
Exposure-related ILD (n=50); HP (n=152); sarcoidosis (n=52); unclassifiable IIP (n=2,577); iNSIP (n=64); other ILD (n=64)
D
Patients included in the MDV database
N=43,154,754
Patients prescribed oral and IV injectable glucocorticoids/ immunosuppressive drugs/anti-CTD drugs for ILD treatment
n=2,035,603
Excluding patients prescribed these drugs before 29 May 2020
n=1,518,712
Patients with extractable baseline data for ≥6 months pre-index (baseline period) and ≥2 months post-index (follow-up period)
n=485,177
Patients with fibrosing ILD ICD-10 disease codes within 6 months before/on the index date
n=19,102
Patients aged ≥20 years on the index date
n=19,071
Excluding patients with only IPF disease codes, without any fibrosing ILD disease codes other than IPF and/or any underlying disease codes within 6 months before/on the index date
Final cohort: N=18,403
Autoimmune-related ILD subgroups:
SSc-ILD (n=599); MCTD-ILD (n=117); PM/DM-ILD (n=824); RA-ILD (n=291); SLE-ILD (n=492); Sjögren ILD (n=678); other autoimmune ILD (n=806)
Non-autoimmune-related ILD subgroups:
Exposure-related ILD (n=332); HP (n=297); sarcoidosis (n=270); unclassifiable IIP (n=12,480); iNSIP (n=294); other ILD (n=2,453)

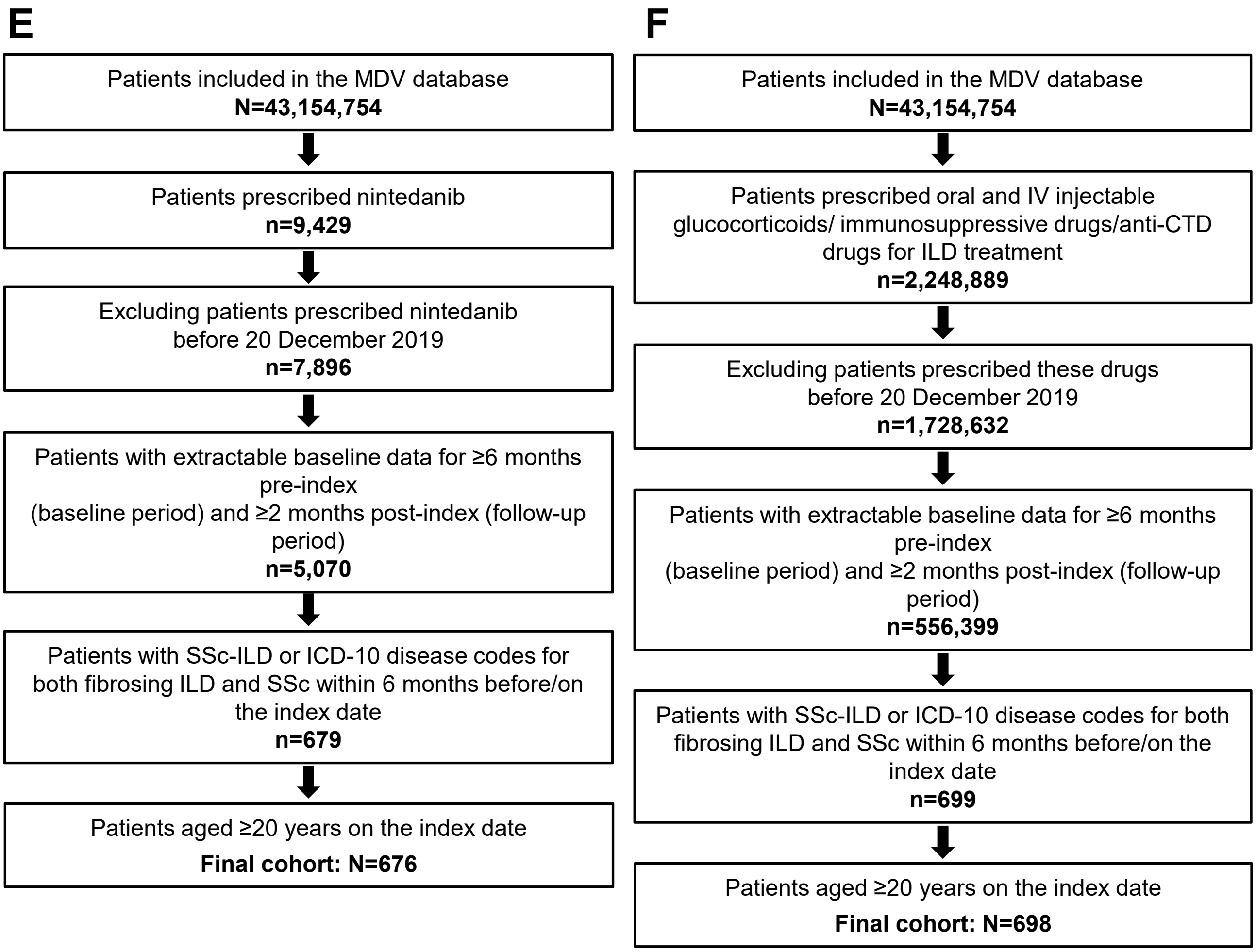


CTD, connective tissue disease; HP, hypersensitivity pneumonitis; ICD-10, International Classification of Diseases-10th Revision; IIP, idiopathic interstitial pneumonia; ILD, interstitial lung disease; iNSIP, idiopathic nonspecific interstitial pneumonia; IPF, idiopathic pulmonary fibrosis; IV, intravenous; MCTD, mixed connective tissue disease; MDV, Medical Data Vision; PM/DM, polymyositis/dermatomyositis; PPF, progressive pulmonary fibrosis; RA, rheumatoid arthritis; SLE, systemic lupus erythematosus; SSc, systemic sclerosis.

**Supplementary Figure S3.** Number of drugs for ILD treatment concomitant with antifibrotics at the time of initiation of antifibrotic treatment by underlying disease.

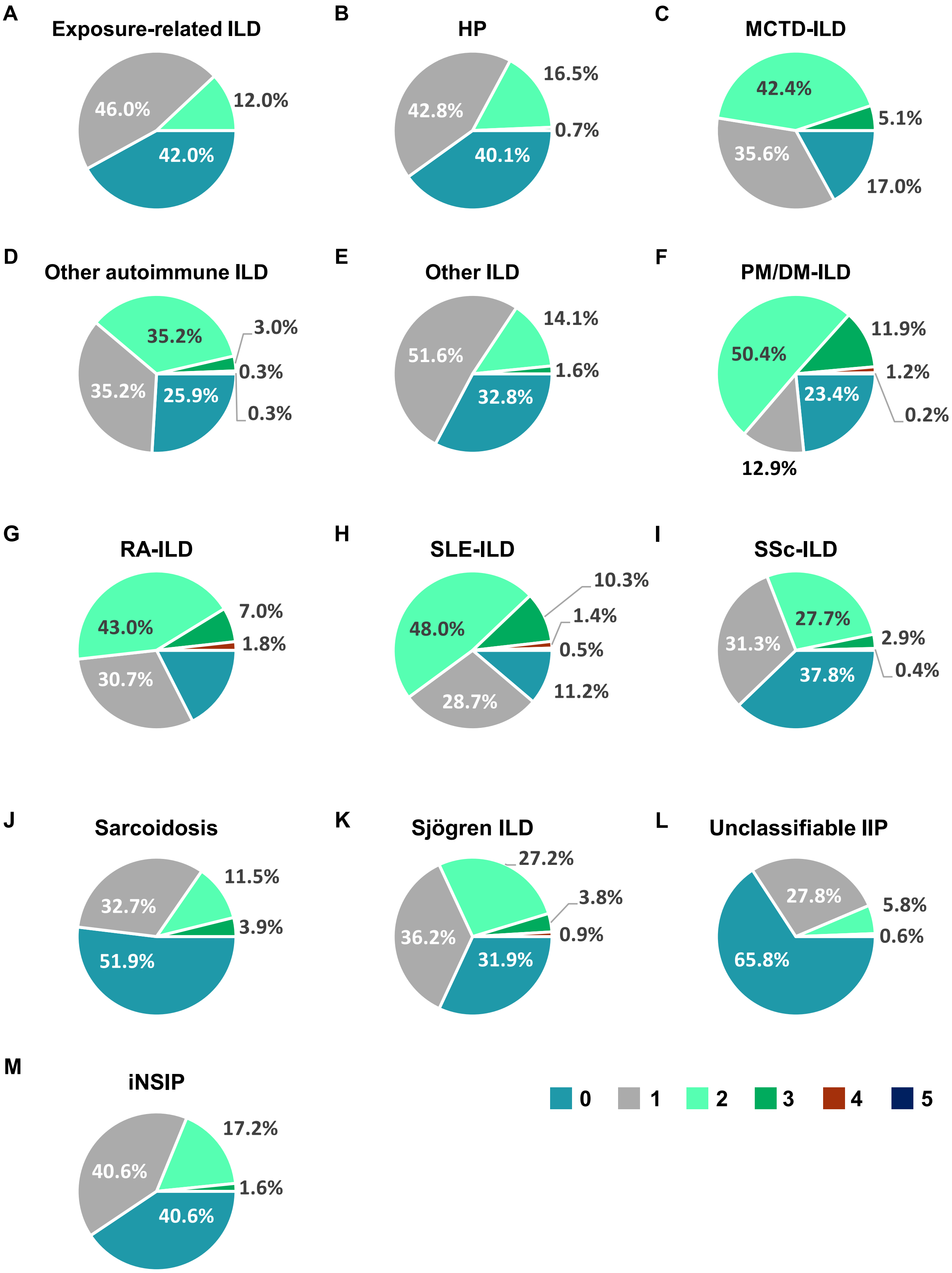

| Statistic | Nintedanib users in patients with PPF | | | | | | |
|---|---|---|---|---|---|---|---|
| | Exposure-related ILD | HP | MCTD-ILD | Other auto-immune ILD | Other ILD | PM/DM-ILD | RA-ILD |
| Mean (SD) | 0.70 (0.68) | 0.78 (0.74) | 1.4 (0.83) | 1.2 (0.89) | 0.84 (0.72) | 1.6 (1.0) | 1.5 (0.92) |
| Median (IQR) | 1 (0–1) | 1 (0–1) | 1 (1–2) | 1 (0–2) | 1 (0–1) | 2 (1–2) | 2 (1–2) |

| Statistic | Nintedanib users in patients with PPF | | | | | |
|---|---|---|---|---|---|---|
| | SLE-ILD | SSc-ILD | Sarcoidosis | Sjögren ILD | Unclassifiable IIP | iNSIP |
| Mean (SD) | 1.6 (0.90) | 0.97 (0.90) | 0.67 (0.83) | 1.1 (0.91) | 0.41 (0.63) | 0.80 (0.78) |
| Median (IQR) | 2 (1–2) | 1 (0–2) | 0 (0–1) | 1 (0–2) | 0 (0–1) | 1 (0–1) |

HP, hypersensitivity pneumonitis; IIP, idiopathic interstitial pneumonia; ILD, interstitial lung disease; iNSIP, idiopathic nonspecific interstitial pneumonia; IPF, idiopathic pulmonary fibrosis; IQR, interquartile range; MCTD, mixed connective tissue disease; PM/DM, polymyositis/dermatomyositis; RA, rheumatoid arthritis; SD, standard deviation; SLE, systemic lupus erythematosus; SSc, systemic sclerosis.

**Supplementary Figure S4.** Number of drugs for ILD treatment prior to the initiation of antifibrotics by underlying disease..

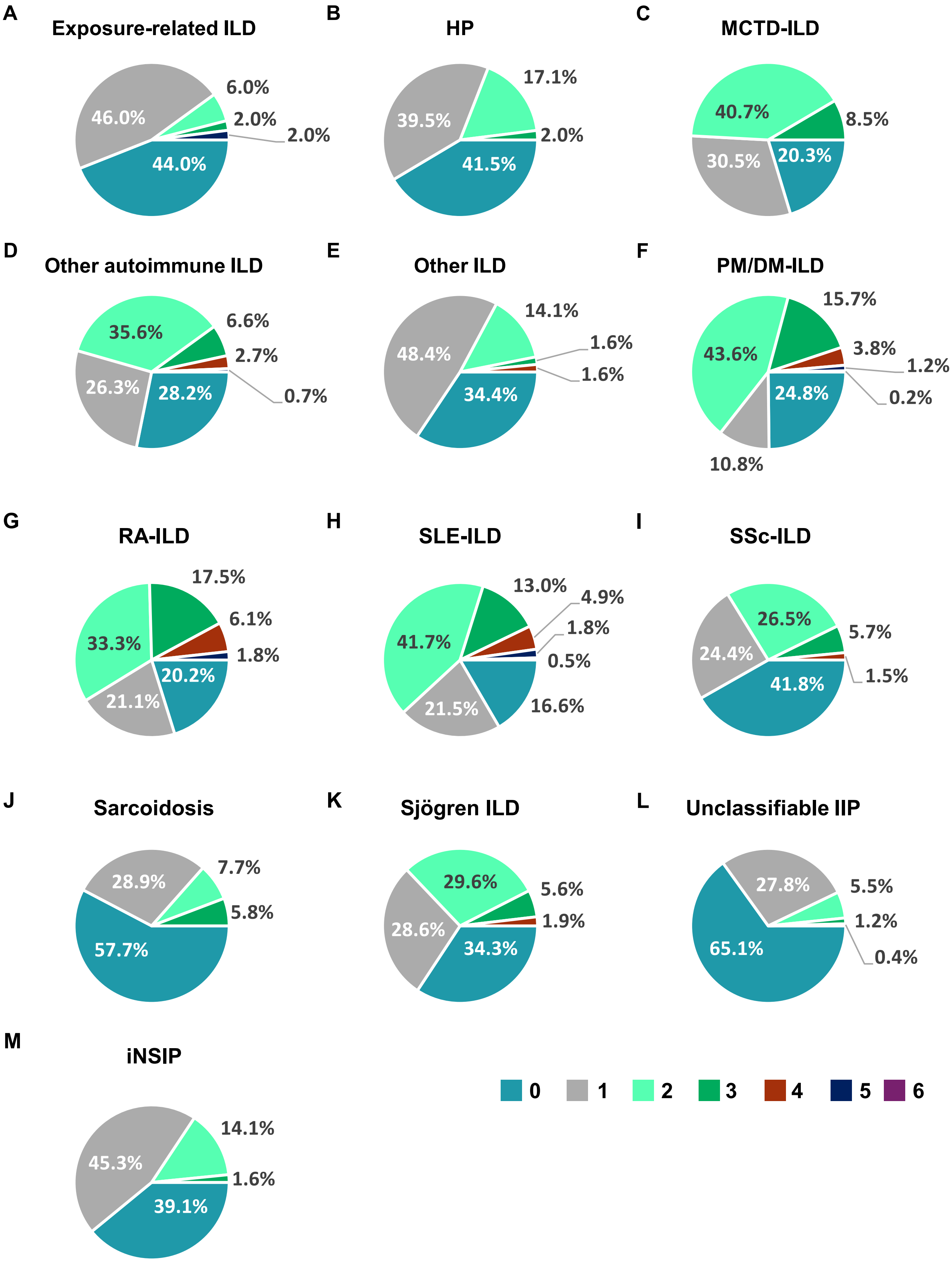

| Statistic | Nintedanib users in patients with PPF | | | | | | |
|---|---|---|---|---|---|---|---|
| | **Exposure-related ILD** | **HP** | **MCTD-ILD** | **Other auto-immune ILD** | **Other ILD** | **PM/DM-ILD** | **RA-ILD** |
| Mean (SD) | 0.74 (0.92) | 0.80 (0.79) | 1.4 (0.91) | 1.3 (1.1) | 0.88 (0.83) | 1.7 (1.2) | 1.7 (1.2) |
| Median (IQR) | 1 (0–1) | 1 (0–1) | 1 (1–2) | 1 (0–2) | 1 (0–1) | 2 (1–2) | 2 (1–2.75) |

| Statistic | Nintedanib users in patients with PPF | | | | | |
|---|---|---|---|---|---|---|
| | **SLE-ILD** | **SSc-ILD** | **Sarcoidosis** | **Sjögren ILD** | **Unclassifiable IIP** | **iNSIP** |
| Mean (SD) | 1.8 (1.2) | 1.0 (1.0) | 0.62 (0.87) | 1.1 (1.0) | 0.44 (0.69) | 0.78 (0.74) |
| Median (IQR) | 2 (1–2) | 1 (0–2) | 0 (0–1) | 1 (0–2) | 0 (0–1) | 1 (0–1) |

HP, hypersensitivity pneumonitis; IIP, idiopathic interstitial pneumonia; ILD, interstitial lung disease; iNSIP, idiopathic nonspecific interstitial pneumonia; IPF, idiopathic pulmonary fibrosis; IQR, interquartile range; MCTD, mixed connective tissue disease; PM/DM, polymyositis/dermatomyositis; RA, rheumatoid arthritis; SD, standard deviation; SLE, systemic lupus erythematosus; SSc, systemic sclerosis.
